# Reirradiation as part of a salvage treatment approach for progressive non-pontine pediatric high-grade gliomas: preliminary experiences from the German HIT-HGG study group

**DOI:** 10.1186/1748-717X-9-177

**Published:** 2014-08-12

**Authors:** Klaus Müller, Heike Scheithauer, Sophie Pietschmann, Marion Hoffmann, Jochen Rössler, Norbert Graf, Brigitta G Baumert, Hans Christiansen, Rolf-Dieter Kortmann, Christof M Kramm, André O von Bueren

**Affiliations:** Department of Radiation Oncology, University Medical Center Leipzig, Leipzig, Germany; Department of Radiation Oncology, University of Munich – LMU, Munich, Germany; Division of Pediatric Hematology and Oncology, Department of Pediatrics and Adolescent Medicine, University Medical Center Goettingen, Goettingen, Germany; Center for Pediatrics and Adolescent Medicine, Clinic IV: Pediatric Hematology and Oncology, University Hospital Freiburg, Freiburg, Germany; Department of Pediatric Hematology and Oncology, Saarland University, Homburg/Saar, Germany; Department of Radiation-Oncology and Clinical Cooperation Unit Neurooncology, MediClin Robert-Janker-Clinic & University of Bonn Med Ctr, Bonn, Germany; Department of Radiotherapy, Hannover Medical School, Hannover, Germany

**Keywords:** Progressive, Relapsed, High-grade gliomas, Pediatric, Reirradiation, Children

## Abstract

**Background and purpose:**

The aim of the present analysis was to assess the feasibility, toxicity, and the tumor control of reirradiation as a salvage treatment for progressive pediatric non-pontine high-grade gliomas (HGG).

**Patients and methods:**

The database of the Reference Center for Radiation Oncology of the German HIT (HIT = German acronym for brain tumor) treatment network for childhood brain tumors was screened for children who were reirradiated for progressive non-pontine HGG.

**Results:**

We identified eight patients (WHO grade III: n = 5; WHO grade IV: n = 3) who underwent reirradiation between April 2006 and July 2012. Median age was 13.5 years at primary diagnosis and 14.8 years at first progression. All patients initially underwent surgery (incomplete resection, n = 7; biopsy, n = 1) followed by radiochemotherapy. Relapses occurred inside (n = 2), at the margin (n = 4), and outside of the preirradiated area (n = 2). In all patients, reirradiation was tolerated well without significant acute toxicity. Temporary clinical improvement and tumor regression on magnetic resonance imaging (MRI) following reirradiation was reported (n = 3). However, all patients finally died by disease progression. Median survival time was 26.2 months from initial diagnosis and 11.4 months after first progression. Median time interval between initial radiotherapy and first reirradiation was 9.0 months. In six patients, all macroscopic tumor deposits were reirradiated. In these patients, median progression-free (overall) survival from the start of reirradiation was 2.4 (4.6) months.

**Conclusion:**

Our analysis, although based on a limited patient number, suggests that reirradiation of progressive non-pontine HGG is feasible in children. Benefit in terms of quality of life and/or survival needs to be assessed in a prospective and ideally in a randomized manner.

## Background

Until present, prognosis of progressive high-grade gliomas (HGG) has remained dismal. Based on numerous retrospective studies reporting on encouraging survival rates and favorable toxicity profile, reirradiation has been widely accepted as an useful therapeutic option for adult patients with relapsed HGG [1–3]. However, as tumor and molecular biology differs between adult and pediatric HGG and as the juvenile brain is particularly vulnerable to radiation-induced injury, results from the above mentioned studies cannot be extrapolated to children [4]. In this analysis we specifically address the feasibility, outcome, and radiation-induced toxicity in a small cohort of pediatric patients with progressive non-pontine HGG who underwent a salvage treatment containing a second course of radiation therapy.

## Patients & methods

The database of the Reference Center for Radiation Oncology of the German HIT (HIT = German acronym for brain tumor) treatment network for childhood brain tumors was screened, between 2006–2012, for children who underwent a second course of radiation therapy for progressive non-pontine HGG. Clinical information was extracted from patient medical charts. The date of initial diagnosis was defined as the date of the first tumor resection or biopsy. The date of progression was determined by neuroradiological imaging. Progression-free (PFS) and overall survival (OS) were assessed using the Kaplan–Meier method. All statistical analyses were performed with SPSS, version 20 (SPSS Inc., Chicago, IL, USA). General informed consent had been given for data acquisition and analyses in the context of the corresponding clinical trials of the first-line treatment.

## Results

We identified eight pediatric HGG patients (male, n = 5) who underwent a second course of radiation therapy between April 2006 and July 2012. Median age was 13.5 years (range, 10.3 – 16.7 years) at initial diagnosis and 14.8 years (range, 11.0 - 17.5 years) at first progression after initial treatment.

### First-line treatment

Surgery included incomplete resection (n = 7), or biopsy (#6). All patients were treated by adjuvant radiochemotherapy (median total dose 59.4 Gy, range, 54.0 Gy – 60.0 Gy) according to the German HIT-GBM D (EU-205100, NCT00278278) (n = 4) or HIT-HGG 2007 (EudraCT 2007-000128-42; ISRCTN19852453) (n = 4) protocols. One patient (#2) received methotrexate (MTX) prior to radiochemotherapy (according to the HIT-GBM D protocol, arm M), and second look surgery of the irradiated tumor was performed. Another patient (#6) received chemotherapy consisting of carboplatin, etoposide and vincristine (VCR) prior to radiochemotherapy as a supratentorial primitive neuroectodermal tumor (CNS-PNET) had been initially suspected before central review confirmed a HGG. All patients received maintenance chemotherapy consisting of temozolomide (TMZ) (n = 5) or lomustine (CCNU), VCR and steroids (n = 3) (Table 1).

**Table 1:** **Patients characteristics I, first-line treatment and time to first progression**

Patient	Age at initial Dx (years)/gender	Tumor location	Pathology	First-line treatment	RT 1 Gy/fractions/concurrent chemotherapy	Maintenance chemotherapy	Time from initial Dx to first relapse (months)
1	11/M	temporo-parietal	AA	pSx → RCT → mCT	54.0/27/ PEV/VCR/PEIV	TMZ	9.5
2	15/M	central	AA/GBM*	pSx → MTX → RCT → second surgery → mCT	59.4/33/ PEV/VCR/PEIV	CCNU/VCR (VBL)	17.4
3	10/F	frontal	AOD	pSx → RCT → mCT	59.4/33/ PEV/VCR/PEIV	CCNU/VCR/prednisolone	9.4
4	13/F	parietal + insular	GBM	pSx → RCT → mCT	60/30/TMZ	TMZ	5.0
5	13/M	frontal	GBM	pSx → RCT → mCT	59.4/33/ TMZ	TMZ	49.9
6	14/M	hemispheric	AA	Bx →CT (carboplatin, etoposide, VCR) → RCT → mCT	54/30/ PEV/VCR/PEIV	CCNU/VCR/prednisolone	12.2
7	10/M	temporal	GBM	pSx → RCT →PD → mCT	59.4/33/ TMZ	TMZ	2.5
8	16/F	thalamic	AA	pSx → RCT →mCT	59.4/33/ TMZ	TMZ	9.0

Dx: diagnosis, M: male, F: female, AA: anaplastic astrocytoma (WHO grade III), AOD: anaplastic oligodendroglioma (WHO grade III), GBM: glioblastoma multiforme (WHO grade IV), Bx: biopsy, pSx: partial surgery, RT: radiation therapy, RCT: radiochemotherapy, mCT: maintenance chemotherapy, TMZ: temozolomide, CCNU: lomustine, PEV: cisplatin, vincristine, CCNU, VCR: vincristine, VBL: vinblastine, PEIV: cisplatin, vincristine, CCNU, ifosfamide, PD: progressive disease. →: followinged by the next treatment.

*AA at initial diagnosis, GBM at first recurrence.

### Histology

Histological assessment initially revealed five WHO grade III (anaplastic oligodendroglioma (AOD) n = 1, anaplastic astrocytoma (AA) n = 4) and three WHO grade IV tumors (glioblastoma multiforme (GBM)). In addition, the tumor tissue from one patient with an AA (#2) showed at the time of the first relapse histopathological features fulfilling the diagnostic criteria of a GBM (AA → GBM). All cases were centrally reviewed (German Brain Tumor Reference Center, Bonn, Germany).

### Second-line treatment

Salvage treatment after first relapse differed considerably between the eight patients.

**Patient #1** (AA) relapsed at the margin of the preirradiated area 9.5 months after surgery. Subsequently, he received stereotactic hypofractionated reirradiation (single doses of 4 × 5 Gy and 1 × 4.2 Gy) to a total dose of 24.2 Gy and concurrent TMZ. Prior exposition to radiotherapy in the reirradiated area was approximately 24 Gy. Salvage treatment was tolerated well without major complications. After reirradiation, two additional cycles of PCV chemotherapy consisting of procarbazine, CCNU and VCR were administered. Three months after the start of reirradiation, a deterioration of the patient’s general condition was observed. A cranial MRI showed clear evidence for a progressive disease. The patient died 4.9 months after the relapse.

**Patient #2** (AA) relapsed at the margin of the preirradiated area 17.4 months after surgery. Prior radiation exposure was approximately 30 Gy. An incomplete tumor resection was performed. Interestingly, whereas the histopathological assessment of the initial tumor revealed an AA, the tissue of the relapsed tumor showed clear evidence for a GBM. Subsequently, a treatment with TMZ (orally) and intraventricular MTX was administrated. Approximately 13 months after the relapse tumor resection cranial MRI demonstrated again a progressive disease. A reirradiation (single dose 1.8 Gy, total dose 55.8 Gy) with concurrent TMZ was performed. Apart from a focal alopecia and moderate erythema no acute radiation-induced adverse reactions or toxicities were reported. Hematotoxicity was mild. Of note, the boy was able to attend the school during the treatment. Following re-radiochemotherapy high-dose TMZ was administered. Regrettably, the child did experience disease progression only 2.4 months after the start of reirradiation. Despite a further salvage treatment attempt with nimotuzumab, the patient died of disease progression 20.5 months after the first recurrence.

**Patient #3** (AOD) relapsed at the margin of the preirradiated area 9.4 months after the surgery. Following partial resection, the girl received reirradiation with 6 × 5 Gy without any complications. Then, the girl received dendritic cell vaccination, 1.6 months after start of the reirradiation, the cranial MRI again revealed disease progression. The child finally died due to tumor progression 5.3 months after the first relapse.

**Patient #4** (GBM) recurred at the margin of the radiation field 5.0 months after the initial surgery. Salvage treatment consisted of reirradiation (17 × 1.8 Gy) with concurrent TMZ, and this treatment was tolerated well. Subsequently, the girl received metronomic chemotherapy with the COMBAT regimen [5]. The tumor responded and the neurological status of the patient significantly improved. Unfortunately, 35.9 months after the start of reirradiation the patient experienced a multilocular second relapse. The girl died 39.5 months after the first relapse despite the girl was treated with a salvage chemotherapy consisting of CCNU and trofosfamide.

**Figure 1:**
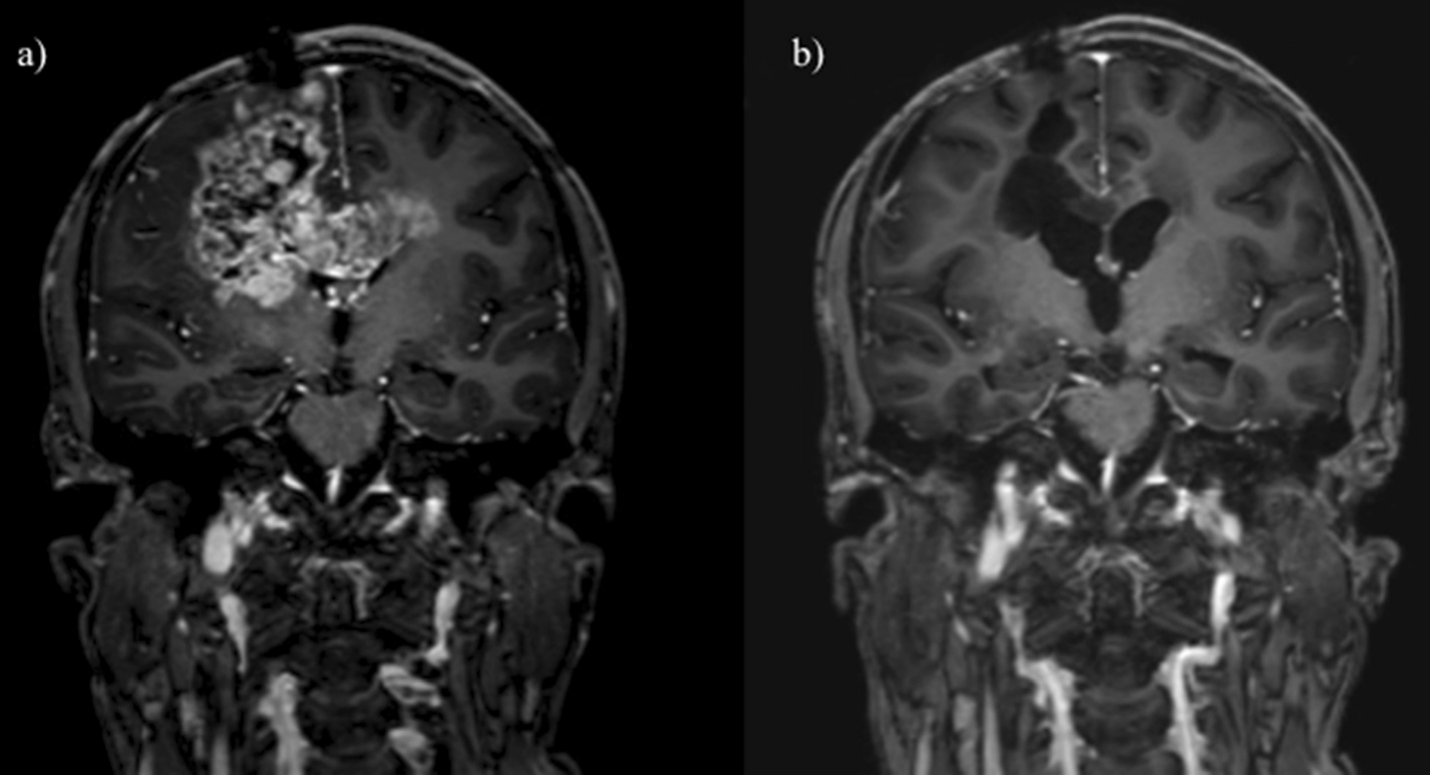
**Patient 5: Contrast enhanced T1 weighted MRI, coronal plane, a) before, b) four weeks after reirradiation: partial remission of the relapsed tumor four weeks after a second course of radiation therapy with 17 × 1.8 Gy = 30.6 Gy.**

**Patient #5** (GBM) experienced a relapse within the preirradiated area 49.9 months after primary surgery. The salvage chemotherapy with TMZ was initiated and this resulted initially in a tumor regression. Due to a second tumor progression the patient finally received reirradiation with 17 × 1.8 Gy. This treatment was tolerated remarkably well, despite a relatively large radiation field due to the large tumor volume of 125 cm^3^. Moreover, the patient received bevacizumab every second weeks. Four weeks after the end of the reirradiation, a cranial MRI showed a very good partial response of the relapsed tumor (Figure 1). At the same time, the neurological status of the boy improved. Unfortunately, 2.9 months after the start of reirradiation, the patient experienced a multilocular tumor progression within and outside of the reirradiated area. Despite an intensification of the ongoing bevacizumab treatment by addition of irinotecan, the patient died 11.5 months after the first relapse.

**Patient #6** (AA) had a multilocular relapse outside of the preirradiated volume 12.2 months after initial surgery. With a palliative objective, he received reirradiation focused on a symptomatic tumor deposit in the left cerebellar peduncle (25 × 1.8 Gy). Metronomic TMZ was administered simultaneously. Treatment was effective. Neurological status, especially the gait pattern, significantly improved. Six weeks after the end of irradiation, cranial MRI demonstrated a tumor regression within and progressive disease outside of the reirradiated area. There were no radiation-induced side effects reported requiring any supportive treatment. Approximately two months later, the boy received radiation therapy for the third time. The radiation therapy was directed at a tumor deposit in the area of the right lateral ventricle (17 × 1.8 Gy). The treatment was well tolerated. Two months after the end of the third irradiation, the MRI again showed disseminated tumor progression, however not in the preirradiated areas. Subsequently, high-dose TMZ (5 days on/5 days off) was started. Finally, a fourth course of stereotactic radiation therapy to a tumor deposit in the right cerebellopontine angle (3 × 5 Gy) was given without any acute side effects. The patient finally died 11.3 months after the first relapse.

**Patient #7** (GBM) relapsed locally 2.5 months after the initial surgery. The relapsed tumor was incompletely resected. The boy received dendritic cell vaccination [6]. The patient finally experienced a multilocular relapse with leptomeningeal spread along the entire craniospinal axis. He subsequently underwent palliative craniospinal irradiation with 10 × 3 Gy with concurrent TMZ. Nevertheless, the disease progressed and the boy finally died 10.6 months after the first relapse.

**Patient #8** (AA) experienced a local relapse, 9.0 months after the initial surgery. The relapsed tumor progressed slowly. In the further course of the disease, a cerebellar metastasis with brain stem infiltration occurred. This tumor manifestation was subsequently irradiated with conventionally fractionated reirradiation to a total dose of 54 Gy. There were no supportive treatments requiring radiation-induced side effects. Shortly after, the patient suffered from a multilocular progression and refused further treatment. She finally died 9.2 months after the beginning of reirradiation and 20 months after first relapse (Table 2).

**Table 2:** **Patients characteristics II, salvage treatment, time from first to second progression and time from initial diagnosis to death**

Patient	Location of first relapse	Salvage treatment and further course of disease	R (C) T 2 (Gy)/fractions/ concurrent chemotherapy	Time from start of second RT to progression (months)	Time from start of second RT to death (months)	Time from first relapse to death (months)
1	margin of RT 1 field	sRCT → PCV → PD → death	24.2/5/ TMZ	3.2	4.6	4.9
2	margin of RT 1 field	pSx (GBM) → CT (MTX + TMZ) → second relapse → RCT 2 → mCT (TMZ) → PD → nimotuzumab → PD → death	55.8/31/TMZ	2.4	6.1	20.5
3	margin of RT 1 field	pSx → RT 2 → PD → dendritic cell vaccination → PD → dendritic cell vaccination + TMZ → PD → death	30/6	1.6	3.8	5.3
4	margin of RT 1 field	RCT 2 → COMBAT chemotherapy → multilocular recurrence → lomustine, trofosfamide → death	30.6/17/TMZ	35.9	38.3	39.5
5	within RT 1 field	TMZ → PD → RT 2 → 30.6/17 → bevacizumab → multilocular recurrence → irinotecan + bevacizumab → death	30.6/17	2.9	6.7	11.5
6	multilocular, outside RT 1 field	RCT 2 (cerebellar peduncle) → mCT (TMZ) → RT 3 (30,6 Gy, lateral ventricle) → PD → TMZ → multilocular progress → TMZ + RT 4 (3 **×** 5 Gy, cerebellopontine angle) → PD → death	45/25/TMZ	not reasonably measurable*	10.3	11.3
7	within RT 1 field	pSx → dendritic cell vaccination → extensive dissemination → RCT 2 (CSI + TMZ) → PD death	30/10 /TMZ	1.4	3.9	10.6
8	outside RT 1 field	watch and wait →local PD and metastasis to cerebellum → RT (cerebellum) → multilocular relapse → further treatment refused → death	54/30	not reasonably measurable*	9.2	20.0

RT: radiation therapy, RCT: radiochemotherapy, sRCT: stereotactic radiotherapy + concurrent chemotherapy, PCV: procarbazine, lomustine, vincristine, PD: progressive disease, TMZ: temozolomide, pSx: partial surgery, GBM: glioblastoma multiforme (WHO grade IV), CT: chemotherapy, MTX: methotrexate, mCT: maintenance chemotherapy, COMBAT: Combined Oral Metronomic Biodifferentiating Antiangiogenic Treatment (including low-dose daily temozolomide, etoposide, celecoxib, vitamin D, fenofibrate and retinoic acid), CSI: craniospinal irradiation. →: followed by the next treatment.

*as only part of the relapsed tumor deposits were irradiated.

### Survival

**Figure 2:**
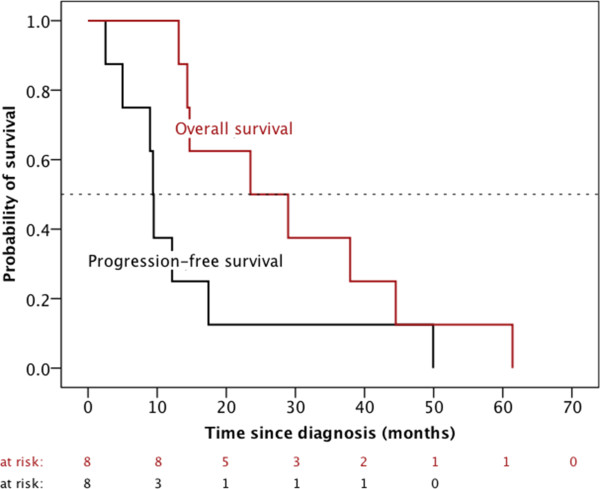
**Progression-free and overall survival after initial diagnosis of 8 children with high-grade gliomas, who underwent a second course of radiotherapy after tumor progression.**

**Figure 3:**
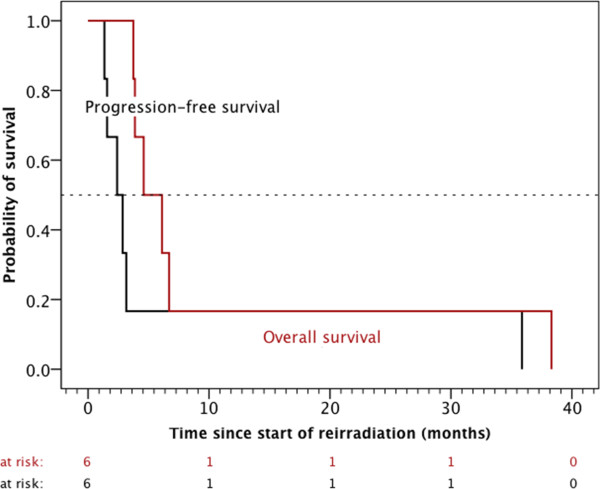
**Progression-free and overall survival after the start of reirradiation of the six children, in whom the planned target volume of reirradiation contained all macroscopic tumor deposits.**

The median survival time from initial diagnosis was 26.2 months (range, 13.1 – 61.4 months) (Figure 2) and 11.4 months (range, 4.9 – 39.5 months) from the time of the first recurrence. Median time interval between the end of the initial radiotherapy and the beginning of the reirradiation was 9.0 months (range, 3.8 – 52.1 months). The tumor control of the six patients, who received reirradiation of all visible tumor lesions was moderate with a median progression-free (overall) survival after the start of reirradiation of 2.4 ± 0.8 months (4.6 ± 1.4 months) (Figure 3).

## Discussion

### General aspects

In the present analysis, we retrospectively assessed eight pediatric patients with non-pontine HGG who underwent reirradiation as part of their salvage treatment after disease progression. Our series is small, however, to the best of our knowledge, this is the first report on reirradiated pediatric non-pontine HGG.

Surprisingly, the idea of reirradiating progressive childhood HGG dates back to the 1980ies. In the Children’s Cancer Group (CCG) study 943, patients who showed tumor recurrences 12 months after the diagnosis could optionally receive irradiation to the recurrent tumor sites up to a dose of 3000 rad in 15–18 fractions. However, in none of the 43 relapsed patients the possibility of reirradiation was exhausted [7]. Apparently, pediatric radiation oncologists have been reluctant to reirradiate relapsed or progressive HGG due to their increased vulnerability of the CNS to radiation therapy. Hence, the fear of an unacceptably high incidence of neurological and other radiotherapy-induced toxicity and sequelae may have limited the use of a second course of radiation therapy. Moreover, even in adult patients, the benefit of reirradiation in terms of survival is still an issue of debate. To assess the impact of reirradiation in adult recurrent GBM patients, the Radiation Therapy Oncology Group (RTOG) 1205 randomized phase II trial is currently validating the survival benefit of reirradiation in a prospective randomized setting.

### Feasibility and safety of reirradiation in children

In our small series, we report on children receiving a second course of radiation therapy. In four cases (patients #1 – #4) the target volumes for reirradiation were located at the margins of and in two cases (patients #5 and #7) even within the former radiotherapy fields. Hence, significant ionizing preloads to the surrounding normal brain tissue ranging between 25 Gy (see patients #1 and #2) and almost 60 Gy had to be expected. Moreover, at least in patient #4, the time interval between the end of initial and the beginning of the reirradiation was rather short (3.8 months). Most experts in the field suggest a radiation-free interval of at least six months between the first and second irradiation [8–10]. However, our very limited experience might provide evidence that some patients may tolerate reirradiation within a shorter (<6 months) radiation-free interval.

The reirradiation was tolerated remarkably well in our small cohort. Alopecia and reddening of the skin were reported. No major unexpected toxicities, and no treatment-related deaths were reported. Moreover, no reirradiation-induced necrosis which would have required treatment did occur in any patient during the further course of the disease. This is in accordance with the conclusions of two important reviews on normal brain tissue tolerance which stated that radiation-induced necrosis has to be expected only at cumulative normalized total doses (single dose, 2 Gy; α/β, 3 Gy) > 102 Gy and just 1–2 years after irradiation, i.e. after the expected survival time in progressive HGG patients [11, 12].

### Tumor response following reirradiation

In three of our patients, temporary but significant improvement of the neurological status and regression of the (re-) irradiated tumor deposits were reported (patient #4, #5, and #6). These findings are in agreement with the experiences reported by Fontanilla and colleagues. They published a series of five children with diffuse intrinsic pontine gliomas whose disease progressed after initial radiochemotherapy and subsequent salvage chemotherapy. A second course of radiotherapy was given with concurrent chemotherapy up to a dose of 20 Gy or 18 Gy. Four patients had substantial clinical improvement of their symptoms (improvement in speech, ataxia, and swallowing). Three patients showed an improved mobility after reirradiation. Four patients had decreased tumor size on posttreatment MRI. Acute radiation-related toxicities were fatigue (n = 2), alopecia (n = 2), and reduced appetite (n = 1). No grade 3 or 4 toxicities were reported [13].

### Impact of reirradiation on survial

Regrettably, in our small series, reirradiation did not demonstrate convincing efficacy in terms of long-term tumor control and survival. In six patients the target volume of reirradiation contained all macroscopic tumor deposits. Only one of these patients (patient #4) showed sustained stable disease for 35.9 months after reirradiation. Surprisingly, the histology of the tumor was a GBM, and the child had progressed only five months after initial diagnosis despite intense first-line treatment consisting of surgery, followed by adjuvant radiochemotherapy (TMZ) and TMZ maintenance chemotherapy. The other five patients did progress less than 3.5 months after the start of reirradiation. Median progression-free (overall) survival from the start of reirradiation was 2.4 ± 0.8 months (4.6 ± 1.4 months) and hence by far less than hoped on the basis of prior experiences gained in adults who had undergone a second course of radiation therapy for refractory HGG. Scholtyssek et al. recently reported a median progression-free (overall) survival of 4.3 (7.7) months in a cohort of 64 adult HGG patients (53 × WHO grade IV, 11 × WHO grade III), who had been treated at progression using a second series of radiotherapy with or without concurrent/subsequent chemotherapy [14]. Other authors even reported much more favorable outcomes with median overall survivals ranging between 9.5 and 11 months [15–17]. Of note, the impact of reirradiation on survival appears to be difficult to interpret in our series as we report on a limited number of patients with differing primary treatment and heterogeneous salvage therapy approaches. In addition, three of our patients (patients #2, #5 and #7) received a second course of radiation therapy at the time of the second relapse thereby influencing the survival data of the present study (Table 2).

## Conclusions

In summary, our findings, although based on a limited patient number, suggest that reirradiation might be feasible in progressive pediatric non-pontine HGG. The recommended radiation dose depends on the location of the relapse, the age of the patients, and the ionizing preloads of the relapse area. Further evaluation of such cases is necessary in order to better delineate feasibility, toxicity, and impact on outcome of this new treatment approach in pediatric non-pontine HGG patients with relapsed tumors.

### Ethical standards

This manuscript is in accordance with the ethical standards laid down in the 1964 Declaration of Helsinki and its subsequent amendments.
